# Recent Advances in the Yeast Killer Systems Research

**DOI:** 10.3390/microorganisms11051191

**Published:** 2023-05-01

**Authors:** Elena Servienė, Saulius Serva

**Affiliations:** 1 Laboratory of Genetics, Nature Research Centre, Akademijos 2, 08412 Vilnius, Lithuania; 2 Laboratory of Nucleic Acid Biochemistry, Institute of Biosciences, Life Sciences Center, Vilnius University, Saulėtekio av. 7, 10257 Vilnius, Lithuania

Biocidic phenotype is common in yeast strains isolated from a variety of natural and industrial habitats. These killer systems confer the hosts with the capability to contend for resources and thus dominate in a certain environmental niche by outcompeting rival yeasts as well as other microorganisms, including fungi and bacteria [1,2,3]. To date, more than a hundred killer yeast species of the *Saccharomyces*, *Candida*, *Hanseniaspora*, *Kluyveromyces*, *Metschnikowia*, *Pichia*, *Torulaspora*, etc., genera have been described [3,4].

The yeast killer trait is often determined by the viral system, consisting of two *Totiviridae* dsRNA viruses, LA and M. The M virus solely encodes the toxin protein, while the host–virus LA provides the capsid protein and RNA-dependent RNA polymerase required for the maintenance and replication of both viruses [5]. The mutual relationship between the different LA and M variants is a long-standing problem, addressed recently by new approaches. Aitmanaite et al. [6] demonstrated the presence of generic mechanisms of *Totiviridae* maintenance in yeast cells, based on comprehensive virus exclusion experiments. Different specificity levels of LA viruses were observed in the maintenance of M dsRNA, leading to suggestions regarding the selfish behavior of M dsRNA and, in turn, the importance of satellite virus M for the cellular amount of LA [6]. Ramirez et al.’s [7] data further extend the specificity issue by providing new insights into the genome organization of yeast *Torulaspora delbrueckii* and *Saccharomyces cerevisiae* dsRNA LBC viruses, uncovered by high-throughput sequencing. Comprehensive analysis of *T. delbrueckii* LBC sequences explains the capability of the LBC virus to maintain M viruses, opening up new horizons in the yeast killer field [7].

Yeasts and their innate dsRNA viruses provide a convenient model system for studying the host–virus interactions. The advancements in various “omics” techniques, such as metagenomics, transcriptomics, proteomics, and lipidomics are exploited for elucidation of the functioning of yeast killer systems [8,9,10,11,12,13,14,15]. Transcriptomic analysis of *S. cerevisiae* yeasts performed by high-throughput RNA-Seq revealed a moderate response to viral dsRNA, suggesting the long-term co-adaptation of killer viruses and host cells [11]. The paper by Ravoitytė et al. [15] reported evidence that the viral dsRNAs also alter the transcriptional profiles of *S. paradoxus* yeasts. The distinct action of dsRNA viruses was observed in the regulation of gene transcription in hosts with different phenotypes, linking the viral infection with metabolism [15]. In agreement with transcriptomics data, proteomics analysis revealed a moderate response of the cells to the viral content and so further substantiated the tight integration of the killer viral system with the essential pathways of the host cells [14]. Specific and intrinsic host cell adaptation as a function of the amount of produced killer toxin was further proved by means of transcriptomic and lipidomic analysis in yet another yeast viral system [16].

The attractiveness of killer yeasts in environmental biotechnology (for biological control of plant pathogens), medicine (antifungal immunotherapy, candidates for treatment of animal and human infections), and the food industry (pest control in the production of cheese and wine) is constantly increasing [3,17]. Diaz et al. [2] summarized the information on killer yeasts, acting as biological control candidates against pre- and postharvest fungal pathogens. The authors pointed to limited studies dealing with killer yeasts with efficient antagonistic activity against preharvest stage plant pathogens. The biocontrol potential of *Wickerhamomyces*, *Saccharomyces*, *Candida*, and *Debaryomyces* killer yeasts against numerous fungal pathogens causing postharvest losses was observed, thus emphasizing a broad range of protective activity and promising results [2].

The evidence of the wide assortment and inherent complexity of yeast killer systems has increased rapidly in the last decade. Clearly, we know a bit about dsRNA viruses inhabited by widely accessible yeasts, mainly of American and, more recently, European origin. However, the vast majority of yeasts inherently originating from the Far East still remain below investigators’ radar. The value of relevant investigations into the Ancient World for the representation of true varieties of killer yeast is therefore evident. Given the available tools and acquired knowledge, many more fascinating discoveries await.

As Associate Editors, we would like to acknowledge all of the contributing authors of the Research Topic “Recent Advances in the Yeast Killer Systems Research” for sharing their enchanting results on fundamental and applied studies of killer yeasts.

## Data Availability

Not applicable.
